# Editorial: Vaccines, Immunotherapy and New Antifungal Therapy against Fungi: Updates in the New Frontier

**DOI:** 10.3389/fmicb.2017.01743

**Published:** 2017-09-08

**Authors:** Carlos P. Taborda, Joshua D. Nosanchuk

**Affiliations:** ^1^Department of Microbiology, Institute of Biomedical Sciences, University of São Paulo São Paulo, Brazil; ^2^Laboratory of Medical Mycology, Institute of Tropical Medicine of São Paulo—LIM53/Medical School, University of São Paulo São Paulo, Brazil; ^3^Departments of Medicine and Microbiology and Immunology, Albert Einstein College of Medicine of Albert Einstein College of Medicine Bronx, NY, United States

**Keywords:** vaccines, immunotherapy, antifungal agents, fungi, editorial

Systemic mycoses are caused by geographically delimitated thermally dimorphic fungi or by classical yeast or molds. Among the thermally dimorphic fungi group, we highlight the human diseases of paracoccidioidomycosis, coccidioidomycosis, blastomycosis, histoplasmosis, and sporotrichosis, the last as cause of subcutaneous mycosis. Although these diseases due to thermally dimorphic fungi are exacerbated by immune suppression, other invasive infections due to yeasts and mold are generally opportunistic. Patients with different degrees of immunodeficiency as a result of AIDS, diabetes, organ transplant, use of immunosuppressive drugs and etc, are at increased risks for developing candidiasis or cryptococcosis (reviewed by Travassos and Taborda, 2017). *Aspergillus fumigatus, Fusarium* spp., and *Penicillium* spp. are, for example, increased in patients undergoing hematopoietic stem cell transplantation for treatment of hematological malignancy (Reviewed by Travassos and Taborda, 2017). This editorial explores several different approaches for combating invasive mycoses and highlights exciting future avenues for study.

Fungi are a major cause of morbidity and mortality on the global stage, and while the pathogenesis of some, such as *Candida* spp. and *Aspergillus* spp. have been deeply investigated, others remain significantly understudied, such as *Paracoccidioides* spp. and *Penicillium* spp. Although the exact number of patients affected by invasive mycoses is unknown, it is estimated that there are over 1.5 million cases annually (Brown et al., 2012; Parente-Rocha et al., 2017; Travassos and Taborda, 2017). The most important tools for control of invasive fungal diseases are systemic antifungal drugs, and certain diseases require months to years of continuous administration. Despite this, there are frequent relapses of some diseases and there are numerous reports of increased drug resistance (Kneale et al., 2016; Parente-Rocha et al., 2017). The high costs of these medications limit their availability to some patients, especially in the developing world. There are four main types of antifungal drugs used for invasive infections: Amphotericin B, flucytosine, azoles, and echinocandins (Aguilar-Zapata et al., 2015; Kneale et al., 2016).

There are new and emerging fungal diseases that challenge the medical community. For example, *Candida auris*, first reported in 2009, is frequently multidrug-resistant (Sarma and Upadhyay, 2017). The mechanisms that lead to antifungal resistance in fungi are highly complex and may include mutation of drug targets, overexpression of the targeted protein, expression of an efflux pump, degradation of the drugs, and pleiotropic drug responses (Parente-Rocha et al., 2017; Scorzoni et al.). Besides multidrug-resistant isolates, the highly potent, broad-spectrum amphotericin B is relatively restricted in use due to its side effects (Parente-Rocha et al., 2017). Due to issues such as drug resistance, toxicities, costs, and prolonged treatment regiments, there is an urgent need for the discovery of new drugs for the treatment of invasive mycoses. Screening of libraries of synthetic small molecules or natural products are exciting and promising methods to identify new drugs (Parente-Rocha et al., 2017). Nanotechnology is also being leveraged to improve the efficacy of traditional antifungal drugs with a particular focus on reducting toxicity, while improving biodistribution and drug targeting (Souza and Amaral). One promising option is the use of the natural polymer of alginate as drug delivery vehicle due to its non-toxicity, biodegradability, high biocompatibility, low cost, mucoadhesiveness, and non-immunogenic properties (de Castro Spadari et al.).

The concept of drug repurposing has led the screening of clinically available compounds for use as new antifungal drugs. The HIV aspartic peptidase inhibitors (indinavir, saquinavir, ritonavir, nelfinavir tipranavir, amprenavir, and lopinavir) also display activity against *Candida* spp. and *Cryptococcus* spp. (Cassone et al., 1999; Cenci et al., 2008). Aspartic-type peptidases participate in essential metabolic events of a fungal cell and help fungi during their interactions with the host (reviewed by Palmeira et al.). Palmeira et al. demonstrated the efficiency of aspartic peptidase inhibitors on the virulence by *Fonsecaea pedrosoi* conidial cells (the causative agent of a subcutaneous mycosis) conidial cells by blocking crucial biological process. The anti-helmithic compound mebendazole also has multiple antifungal effects on *Cryptococcus neoformans*, a neurotropic fungus (Joffe et al.). Notably, mebendazole achieves levels in the brain that have antifungal activity against phagocytized *C. neoformans* and the yeasts cells within cryptococcal biofilms as well as causes marked morphological alterations in the yeast cell (Joffe et al.).

Novel compounds have also been explored for their efficacy against fungi. For example, metal-based drugs are being studied due to their therapeutic potentials for diverse pharmacological applications (reviewed by Granato et al.). In this context investigators have analyzed the effect of 1,10-phenanthroline-5,6-dione (phendione) and its metal-based derivatives on *Phialophora verrucosa* conical cells (an agent of chromoblastomycosis, a subcutaneous mycosis) and *in vitro* tests have shown that phendione and its Ag^+^ and Cu^2+^ complexes represent a promising antifungal agent against *P. verrucosa* (Granato et al.).

A synthetic compound previously explored for its cancer chemotherapeutic activities, biphosphinic cyclopalladate C7a, has been tested against several microorganism and parasites, such as *Trypanosoma cruzi, Paracoccidioides brasiliensis, P. lutzii, C. neoformans*, and *C. albicans* (reviewed by Muñoz et al.). Here the authors have demonstrated that C7a is effective *in vitro* against different isolates of *Candida*, including azoles resistant strains (Muñoz et al.).

Genetic manipulation has also been explored as another option for controlling mycoses. For example, the inteins, invasive genetic elements that occur as intervening sequences in conserved coding host genes, are being explored as a new drug target against fungi as *Candida* ssp. (Fernandes et al.).

Antifungal drugs are the basis of systemic mycoses treatment of patients and, an in-depth understanding of the molecular mechanisms underlying their efficacy provides insights into fungal pathogenesis (Ding et al.). Immunosuppression may interfere with chemotherapy efficiency (Travassos and Taborda, 2017). Antifungal vaccines may boost the immune system and enhance the protective effect of antifungal drugs, which allows for a reduction in the time required for treatment and prevention of relapse (Travassos and Taborda, 2017). There is no licensed vaccine for the prevention or treatment of human mycoses. Albeit, there are some groups around the world involved with different strategies for vaccine development or immunotherapy using monoclonal antibodies against systemic mycosis.

Protection against most mycoses involves the activation of the cellular immune response through CD4^+^ T helper cells. T-helper (Th) 1 or Th17 responses may be cytotoxic or involve the secretion of inflammatory cytokines such as IL-12, IL-17A, IFN-γ, GM-CSF, and TNF-α, which active different cell populations as neutrophils, macrophages, and dendritic cells (Parente-Rocha et al., 2017). The progression of fungal infection is related to a decrease in Th1-type response and an increase in the response mediated by CD4^+^ T-helper cells type 2 (Th2), producing cytokines such as IL-4, IL-5, and IL-10. Although the Th2-type response is associated with aggravation of fungal infections, cytokines produced are essential for the control of exaggerated inflammatory responses (Cutler et al., 2007).

The production of vaccines from proteins (peptides) or polysaccharides is a standard approach to vaccination (Travassos and Taborda, 2017). The use of peptides as vaccines has many advantages: they are free of infectious material, can be produced in large scale; include multiple determinants or epitopes; can be modified by lipids, carbohydrates or phosphate, acetyl and terminal amide groups to increase their stability, immunogenicity and solubility; and may be covalently or non-covalently linked to macromolecules for increased immunogenicity (Purcell et al., 2007). Peptide delivery using different formulations is a challenge for the creation of an efficient vaccine. Dendritic cells are very important for both innate and adaptive immune response and play a significant role in the immune response to dimorphic fungi (Thind et al., 2015). Dendritic cells are up to 1,000-fold more efficient in activating T cells than traditional adjuvants. The use of dendritic cells primed with peptide 10 (P10), derived from the *P. brasiliensis* glycoprotein 43 (gp43), as prophylactic or therapeutic vaccine in experimental model using infected mice with yeast cells from *P. brasiliensis* reduces lung fungal burdens (Magalhães et al., 2012). Using a similar approach, Silva et al. utilized dendritic cells primed with P10 in combination with trimethoprim-sulfamethoxazole administration to treat immunocompromised mice infected with *P. brasiliensis*. The authors observed P10-pulsed dendritic cells with or without antifungal drugs are potently effective in combating invasive paracoccidiodomycosis.

During the infection, fungi induce the production of a heterogeneous population of polyclonal antibodies and, individually, these antibodies may increase or decrease protection against fungal infections, as well as may have no effect at all. Since fungi can induce the production of protective antibodies, several studies have shown that these molecules can act as efficient vaccines in the fight against systemic infections caused by fungi such as aspergillosis (Chaturvedi et al., 2005), choroblastomycosis (Nimrichter et al., 2004), candidiasis (Coleman et al., 2009), cryptococcosis (Taborda et al., 2003), paracoccidioidomycosis (Buissa-Filho et al., 2008), and histoplasmosis (Nosanchuk et al., 2012) among others. The main advantage of administering humanized antibodies is that they may have fewer side effects compared to chimeric or non-human antibodies. As an example, a genetically engineered mAbP6E7 antibody against a 70-kDa *Sporothrix* antigen effectively decreased fungal burdens of *S. schenckii* in infected mice (de Almeida et al.).

The expansion of knowledge in mycology obviously is not phenomenon restricted to human or animal pathogens. For instance, the fungi play an extremely important function of the plantae kingdom. The identification of a new strain the can cause wheat stripe rust (Zheng et al.) and the hypovirulence of *Sclerotium rolfsii* caused by association of RNA mycoviurs (Zhong et al.) underscore their impact and the efforts underway to understand their biology.

In sum, the articles in this Frontier's topic broadly paint the spectrum of investigations on new antifungal drugs, host-pathogen interactions and provide a review of the state-of-the-art in vaccinology, immunotherapy, and chemotherapy against fungi. The information presented also underscores areas ripe for future study and details several promising improved therapeutics and therapeutic approaches against fungal invaders.

## Author contributions

All authors listed have made a substantial, direct and intellectual contribution to the work, and approved it for publication.

### Conflict of interest statement

The authors declare that the research was conducted in the absence of any commercial or financial relationships that could be construed as a potential conflict of interest.
